# A Case Study of Hypnosis Enhanced Cognitive Therapy for Pain in a Ventilator Dependent Patient during Inpatient Rehabilitation for Spinal Cord Injury

**DOI:** 10.3390/jcm12134539

**Published:** 2023-07-07

**Authors:** Amy J. Starosta, Katherine S. Wright, Charles H. Bombardier, Faran Kahlia, Jason Barber, Michelle C. Accardi-Ravid, Shelley A. Wiechman, Deborah A. Crane, Mark P. Jensen

**Affiliations:** 1 Department of Rehabilitation Medicine, University of Washington, Seattle, WA 98195, USA; wright6@uw.edu (K.S.W.); chb@uw.edu (C.H.B.); fkahlia@uw.edu (F.K.); wiechman@uw.edu (S.A.W.); dacrane@uw.edu (D.A.C.); mjensen@uw.edu (M.P.J.); 2 Department of Neurological Surgery, University of Washington, Seattle, WA 98195, USA; barber@neurosurgery.washington.edu; 3 Department of Physical Medicine and Rehabilitation, University of Utah, Salt Lake City, UT 84132, USA; michelle.accardiravid@hsc.utah.edu

**Keywords:** pain, spinal cord injury, case study

## Abstract

Early, acute pain following spinal cord injury (SCI) is common, can negatively impact SCI rehabilitation, and is frequently not responsive to biomedical treatment. Nonpharmacological interventions show promise in reducing pain for individuals with SCI. However, most psychological interventions rely heavily on verbal interaction between the individual being treated and the clinician, making them inaccessible for individuals with impaired verbal output due to mechanical ventilation. This case study aims to describe the adaptation and implementation of hypnotic cognitive therapy (HYP-CT) intervention for early SCI pain in the context of mechanical ventilation dependence and weaning. The participant was a 54-year-old male with C2 AIS A SCI requiring mechanical ventilation. Four sessions of HYP-CT were provided during inpatient rehabilitation with assessment prior to intervention, after the intervention sessions, and prior to discharge. The participant reported immediate reductions in pain intensity following each intervention session. Overall, he reported increases in self-efficacy and pain acceptance. He did not report any negative treatment effects and thought the intervention provided support during mechanical ventilation weaning. During treatment, he discontinued opioid pain medications and reported actively using intervention strategies. Our results support the potential for early, hypnotic cognitive therapy for individuals with SCI experiencing pain or distress while dependent on mechanical ventilation.

## 1. Introduction

Chronic pain is a common issue following spinal cord injury (SCI), with reviews estimating that 40% to 80% of individuals with SCI report chronic pain [1] and that this pain is severe in 32% to 53% of those with SCI [2]. People with SCI can experience multiple types of pain simultaneously, including musculoskeletal, neuropathic, and visceral pain [3]. Nociceptive pain is pain that results from activation of (otherwise healthy) nociceptors related to tissue damage or the risk for tissue damage (e.g., muscle overuse) that can happen in any population. In contrast, neuropathic pain is associated with damage to neurons in the central nervous system (CNS: brain or spinal cord) or periphery that continues after an injury heals. In individuals with SCI, such pain is often experienced at or below the level of the SCI [3]. Both types of pain are common following SCI. A 2021 meta-analysis of SCI pain prevalence reported that 58% of people with SCI have neuropathic pain and 45% have nociceptive pain [4].

For many people with SCI, pain starts early following injury and adversely affects SCI rehabilitation. Siddall et al. found that at two weeks post-injury, 90% of people report pain [5]. Those with greater acute pain receive less time in therapy sessions during inpatient rehabilitation and need more modifications to therapy activities [6]. In many cases, more intense acute SCI pain predicts greater chronic pain [5,7]. When pain becomes chronic it is associated with negative outcomes such as depression, sleep disturbance, poorer physical, psychological, social, and occupational function, as well as lower quality of life in people with SCI [8,9,10].

Although longitudinal studies demonstrate the high prevalence of both early and chronic SCI pain, less is known about the factors that contribute to the development of pain after injury. Thus far, most pain prediction research has used a biomedical framework and has therefore focused on biomedical predictors. Although a number of biological predictors of SCI-related pain have been identified, such as sensory hypersensitivity [11], chronological age and sensory and motor preservation [12], and injuries due to gunshot wounds [13], these factors do not fully explain chronic pain intensity or interference. One reason that biological variables alone do not fully explain the development of chronic pain is that all chronic pain, including SCI-related pain, is influenced by psychological and social factors [14]. Dating back to at least the 1960s, pain clinicians and researchers have recognized that pain has multiple components including cognitive/evaluative and affective/emotional, as well as sensory/nociceptive elements [15]. Additionally, biological, psychological, and social factors influence chronic pain and pain behaviors [14]. The biopsychosocial model has been advanced as the way to understand and treat SCI-related pain by leading SCI pain researchers [7,9,16,17,18,19].

Despite the call to conceptualize pain and pain treatment from a biopsychosocial model, management of SCI-related pain continues to be primarily pharmacological. Unfortunately, SCI-related pain is often not responsive to pharmacological intervention. Optimal pharmacological treatment (often involving a combination of anticonvulsants, antidepressants, opioids, or antispasmodics) typically results in only about one third of people experiencing at least a 50% reduction in their pain [9]. Not only do medications have limited efficacy, commonly used pain medications, such as opioids, have negative side effects [8,20], For years, experts have called for research on nonpharmacological treatments for acute SCI-related pain to lessen the development of chronic pain and improve the quality of life [7,21]. In comparison to pharmacological interventions, nonpharmacological interventions have very few negative side effects, and have been found to reduce chronic pain in non-SCI populations [14].

One factor that has consistently been associated with pain intensity for individuals with chronic pain, including individuals with SCI-related chronic pain, is a tendency to focus on negative automatic thoughts about pain, also referred to as pain catastrophizing [22]. Individuals who have this tendency are more likely to experience increased pain intensity, and to have higher levels of physical disability, psychological distress [23], depression [24], and sleep disruption [25]. This issue is particularly relevant for SCI-related pain, as individuals with SCI may experience more pain catastrophizing than other chronic pain populations [26]. A tendency to catastrophize about pain may develop early following SCI and is related to long-term pain intensity and unpleasantness [27], suggesting that these cognitive appraisals about pain are potentially an important intervention target for any chronic SCI pain treatment.

The most common clinical approach for addressing pain catastrophizing is cognitive restructuring (also known as cognitive therapy; CT). This strategy focuses on teaching individuals to become aware of their thoughts about pain, evaluate the impact of those thoughts on their mood and behaviors, and—for those thoughts deemed to have negative effects—develop alternative thoughts that support improved mood or increased engagement with valued activities. Recent reviews recommend the use of CT strategies to manage SCI-related pain mostly based on evidence that CT can reduce pain interference, especially in the areas of sleep and mood [28,29,30]. Taken together, CT has a strong theoretical basis, evidence of some positive effects in SCI pain management, and merits further research.

Another promising nonpharmacological treatment for chronic pain is clinical hypnosis, defined as, “A state of consciousness involving focused attention and reduced peripheral awareness characterized by an enhanced capacity for response to suggestion” (p. 32) [31]. Clinical hypnosis usually involves two steps. First, a hypnotic “induction”, which is designed to enhance responses to clinical suggestions. This is then followed by clinical suggestions, which are provided to engender changes in sensations, mood, or behavior. These suggestions differentiate hypnotic interventions from interventions including relaxation or clinical meditation, which also may be promising interventions for reducing pain interference for individuals with SCI [32]. Traditionally, the clinical suggestions for pain management focus on decreased pain, increased comfort, and improved ability to ignore or be distracted from pain, and/or changing the quality of pain. The last suggestion provided usually involves a “post-hypnotic” suggestion that the benefits experienced by the individual will last beyond the session. Individuals who receive hypnosis treatment may be given recordings of the treatment sessions in order to practice hypnosis on their own between treatment sessions, and also to perform self-hypnosis following a basic self-induction procedure and giving themselves helpful hypnotic suggestions. A recent meta-analysis found that for non-headache pain, hypnosis had a moderate beneficial effect on pain intensity compared to control conditions [33]. There is also evidence that hypnosis might be particularly effective in treating neuropathic pain, which is common among people with SCI [34].

In addition to hypnosis being an effective stand-alone treatment, there is substantial evidence that, when combined with other evidence based cognitive and behavioral therapies, hypnosis enhances the benefits of these treatments. Two meta-analyses—one published in 1985 and a second updated analysis published more recently—of studies that added hypnosis to cognitive and behavioral therapies for pain, insomnia, obesity, or hypertension concluded that the average effect size for the combined treatment was in the large range (e.g., Cohen’s *d* = 1.36) [35,36]. Building on these findings, a hypnosis-enhanced cognitive therapy intervention (HYP-CT) was developed to target negative thoughts about chronic pain for people with disabilities and chronic pain. Initial pilot studies and follow-up clinical trials have consistently found HYP-CT to be effective, resulting in a marked reduction in pain intensity compared to pain education, CT, or hypnosis alone [37,38,39].

These psychological interventions rely heavily on collaboration with the individual receiving the treatment, which can be more difficult for those individuals who are unable to voice due to mechanical ventilation needs. As many as two thirds of individuals with new cervical-level SCI will experience respiratory complications during the acute phase of their injury, often requiring mechanical ventilation [40]. Patients requiring ventilation are a uniquely vulnerable group, as respiratory dysfunction is a major cause of both mortality and morbidity in SCI [40]. Anxiety is very common among those requiring mechanical ventilation [41], and prolonged anxiety and distress may predispose these individuals to more long-term negative psychological outcomes, including depression and posttraumatic stress disorder [42].

Despite the high need for psychological support and intervention for patients requiring mechanical ventilation, there is limited information on how effective psychological interventions are for this population. One case study reported positive results from HYP-CT for an individual with a high-level SCI who was dependent on mechanical ventilation in an outpatient setting [43]. After eight sessions, this individual showed clinically meaningful improvements in sleep quality and pain acceptance, and reductions in pain intensity, interference, and catastrophizing; these improvements were maintained for over a year. 

All of the available evidence in individuals with SCI is from interventions offered on an outpatient basis after chronic pain has already developed. However, as previously discussed, studies have found that chronic pain often begins early in the course of SCI [6,44,45]. There is untapped clinical potential to reduce the negative impact of pain in this uniquely vulnerable population by intervening early in rehabilitation. 

In order to provide early nonpharmacological SCI pain treatment, the previously evaluated CT and hypnosis intervention (HYP-CT) was adapted to be administered during inpatient rehabilitation following SCI. During the recruitment for this feasibility trial, an individual requiring mechanical ventilation was interested in participating in the intervention. This patient met all eligibility requirements, but due to the need to adjust the intervention to meet the patient’s communication needs, he was not enrolled in the primary pilot project. Rather, the aim of this case study is to describe the process of adapting and implementing HYP-CT for early SCI pain in the context of mechanical ventilation-dependence and weaning. 

## 2. Case Study

The participant is a 54-year-old Filipino man who was initially admitted to the intensive care unit (ICU) after an unwitnessed fall down a flight of stairs. He presented with polytrauma, including multiple facial fractures, wrist fractures, and a new onset spinal cord injury. The initial exam using the International Standards for Neurological Classification of SCI (ISNCSCI) on the day after his injury and surgery showed complete sensorimotor tetraplegia, C2, American Spinal Injury Association (ASIA) Impairment Scale (AIS) grade A, although 3 weeks later when he was being admitted to inpatient rehabilitation, his SCI was graded as C1 AIS B SCI. His spine injuries were treated with C2-5 posterior spinal instrumentation and fusion (PSIF) and C3-4 laminectomies. He required mechanical ventilation due to the level of his SCI. Both a tracheostomy and percutaneous endoscopic gastrostomy (PEG) tube were placed on hospital day 6. His course was complicated by ventilator-associated pneumonia. After 16 days of ICU management, the participant was medically stable and ready for an inpatient rehabilitation program. 

Upon transfer to the inpatient rehabilitation unit on hospital day 17, the participant required total assistance for all aspects of self-care and functional mobility. During inpatient rehabilitation, he was actively engaged with therapy teams. He received 15 h of therapy per week, and his therapist rated his participation as “good” to “very good” on the Pittsburgh Rehabilitation Participation Scale (PRPS). On hospital day 13, he began weaning from mechanical ventilation via T-piece spontaneous breathing trials (SBTs) prior to his admission to inpatient rehabilitation and continued SBTs on inpatient rehabilitation. When the cuff was inflated, he communicated by mouthing words, using head nods/shakes, and occasional use of an eye-gaze board. He made steady positive progress and was independent from the ventilator for a full 24 h on hospital day 29. Post-wean progress was complicated by a lower lobe collapse on day 31 but was successfully managed with chest physiotherapy. During the weaning process, the participant continued to be mostly unable to voice due to cuff inflation. As the weaning protocol progressed, he was able to communicate at the 2–3-word level. He was decannulated on hospital day 52, at which point he was able to communicate fully with mild voice impairments mostly due to poor respiratory support. The participant was discharged home with his family 69 days after his injury and admission to the ICU. 

The participant was followed by the Rehabilitation Psychology team throughout hospitalization, first by a clinical psychologist on the acute care consult service, and then by one of the authors (KSW), who is a clinical psychologist on inpatient rehabilitation. The participant denied any history of mental health diagnoses or treatment prior to this hospitalization. He noted that he was a generally positive person and had great support from family and friends. His significant other (SO) and adult son described him as “happy, loving, good humored, funny”, and the “type of person who will do anything for anyone”. Unfortunately, due to the hospital policy for COVID restrictions at the time, the participant was unable to have regular visitors at bedside initially. However, in preparation for discharge, his SO and his son were able to be with him in person to learn about his care and to participate in hands-on training, which the participant reported was very helpful for his mood. 

At baseline assessment, the participant reported significant pain in his neck, chest, and throughout his back (average intensity = 4/10, worst intensity = 8/10). Upon initial presentation, pain was primarily musculoskeletal in nature. The participant reported that pain was often distressing to him and sometimes made it difficult for him to fall asleep. He reported that he felt the worst pain during turns with nursing staff, which occurred every two hours to protect his skin. 

## 3. Methods

### 3.1. Intervention

The intervention was adapted from a standardized outpatient hypnotic cognitive therapy (HYP-CT) [38] protocol to fit into the busy schedule of the inpatient rehabilitation setting. Full descriptions of the intervention and pilot have been published elsewhere [46]. In brief, the intervention utilizes focused attention and perceived automaticity resulting from hypnosis to enhance the efficacy and extend the duration of the positive effects of CT. The primary goals of HYP-CT are to (1) increase the individual’s comfort with ambiguity about the meaning of pain in order to reduce pain catastrophizing, (2) encourage the belief that the individual can gain control over pain and its impact, or self-efficacy, and (3) automatize the process of cognitive restructuring. The intervention sessions are described in Table 1. 

### 3.2. Measures

The same outcome domains that were assessed in the pilot trial were administered (see Table 2) prior to the start of the intervention (baseline), at each intervention session, and prior to discharge (follow-up). A shortened post-discharge assessment was conducted during an outpatient Rehabilitation Medicine appointment 5.5 months after discharge (post-discharge). Full descriptions of all measures collected are published elsewhere [46], while a brief description of the measures relevant to the present case are provided below. 

#### 3.2.1. Demographic and Descriptive Measures

*Demographic variables*: Demographic information was collected using self-report including sex, race, ethnicity, education, employment status, and marital status.

*Pain type*: The pain type was classified as neuropathic or nociceptive based on the participants’ responses to the Spinal Cord Injury Pain Instrument (SCIPI) [47]. The 4-item SCIPI short form is a screening tool that assesses the quality and features of the individual’s worst pain in the past 7 days. Evidence supports its sensitivity and specificity for classifying neuropathic versus nociceptive pain types [47].

*Pain locations*: Information about each pain location was collected at baseline using Version 2.0 of the International SCI Pain Data Set [48]. The participants were asked to rank their three worst pain locations and provide information about each pain. The location, type of pain, intensity, duration, and treatment was collected for each of the three unique pain problems. The average pain intensity in the past week for each pain problem was rated on a 0–10 NRS from 0 = “No pain” to 10 = “Pain as bad as you can imagine”. 

#### 3.2.2. Patient-Reported Outcome Measures

*Treatment satisfaction*: The Benefit, Satisfaction, and Willingness to Continue (BSW) scale was used to assess treatment satisfaction at discharge. The BSW is a patient-reported outcome that reflects the individuals’ perception of the experience and effects of treatment. The question format for each of the 3 items was similar, with a yes/no question asked first; for example question 1 is “Did you receive any benefit from this treatment?” followed up with a question that rates the degree of benefit, satisfaction, or willingness to continue, for example “If yes, have you had little benefit or much benefit?” The BSW measure has shown validity for other symptom-based conditions including overactive bladder [49].

*Global improvement*: The Patient Global Impression of Change (PGIC) was used to assess perceived global improvement at discharge. The PGIC is a 7-point Likert scale on which participants describe how much better or worse they are now compared to the beginning of the intervention. The patients were asked to rate their overall status from the start of the study using the following scale: (1) very much improved; (2) much improved; (3) minimally improved; (4) no change; (5) minimally worse; (6) much worse; (7) very much worse. The PGIC is a standardized outcome measure for SCI clinical research and is often used as a secondary outcome measure [50].

*Adverse events*: At the beginning and end of intervention sessions, the study clinician asked participants if they experienced any adverse events between or during sessions. Adverse effects were also assessed at discharge by the research assistant. 

*Average and worst pain intensity*: The average and worst pain intensity were assessed at baseline and discharge using the 0–10 Numerical Rating Scale (NRS). The participants were asked to rate their worst and average pain intensity in the past week from 0 “No pain” to 10 “Pain as bad as you can imagine”. The NRS is recommended as a core outcome measure in pain studies [51] and has demonstrated validity as a measure of pain intensity through its strong association with other pain measures as well as its ability to detect changes in pain with treatment [52].

*Immediate effects of the treatment sessions on pain*: Prior to the start of each of the four intervention sessions, the current pain intensity was assessed on a 0–10 NRS, and reassessed at the end of the intervention session. In addition, the participants were asked to rate how much relief they experience during the session on a 0–10 scale, with 0 indicating “No relief” and 10 indicating “Complete relief”. 

*Pain interference*: The PROMIS Pain Interference Short Form (PROMIS PI) assesses the impact of pain with questions relevant to people during hospitalization for a traumatic injury [53]. The PROMIS PI has demonstrated validity in diverse clinical populations [54]. Higher scores indicate more pain interference. In order to reduce the assessment burden at post-discharge follow-up, a single pain interference from the Medical Outcomes Study SF-36 [55] was used. This item assesses pain interference on a 1–5 scale, with response options of “not at all”, “a little bit”, “moderately”, “quite a bit”, and “extremely”. Responses can be transformed to a 0–100 score; however, to reduce direct comparisons to PROMIS PI scores, we elected to leave response in categorical format. 

*Pain catastrophizing*: The Concerns about Pain (CAP) scale was used to assess pain catastrophizing at baseline and discharge. It was developed using item-response theory as well as expert and patient feedback [56] and has strong psychometric properties [57]. It is specifically designed to assess negative cognitive responses to anticipated or current pain [57]. The participants were asked how often they had each thought about pain using a response set rated from 1 “Never” to 5 “Always”. Higher scores indicate more pain catastrophizing.

*Pain self-efficacy*: The 2-item short form of the Pain Related Self-Efficacy (PRSE) scale was administered at baseline and discharge [53]. It is an item-response theory measure designed to assess an individual’s belief in their ability to accomplish tasks and activities despite their pain. Participants were prompted to rate their confidence about each item on a scale from 1 “not at all” to 5 “very much”. Higher scores indicate more self-efficacy.

*Pain acceptance*: The Chronic Pain Acceptance Questionnaire-Revised (CPAQ-R) is a 20-item questionnaire that measures pain acceptance through two distinct factors: activity engagement and pain willingness [58]. Participants were asked to rate the truth of each of the twenty statements as it applied to them on a 7-point scale from 0 “never true” to 6 “always true”. Items are summed for a total score, with higher scores indicating higher levels of acceptance.

*Sleep disturbance*: Sleep disturbance was assessed at baseline and discharge using the PROMIS sleep disturbance short form [59] which consists of 8 items assessing problems related to sleep quality over the past 7 days. The individual is prompted to rate each item on a 5-point scale of 1 “never” to 5 “always”. Higher scores indicate more sleep disturbance.

## 4. Findings

### 4.1. Changes in Study Outcome Variables

#### 4.1.1. Pre- to Post-Session Effects

Pain intensity was assessed before and after each session. For all four sessions, the participant reported stable or reduced pain intensity from before to after hypnosis (see Table 3). It is important to highlight that these improvements are considered clinically significant, as they all exceed the suggested 1.6 point clinically significant cut off [60]. Additionally, the participant consistently reported positive responses to the hypnosis, and experiencing “complete relief” during two of the sessions. The participant denied any negative effects associated with the hypnosis treatment throughout the intervention time period. 

Prior to the start of intervention, the participant was receiving an average 8.5 mg of oxycodone daily. He began to use his prescribed 5 mg PRN dose of oxycodone less and less frequently over a 3-week period and had self-weaned from opioids completely by session 3 (see Table 4). The patient continued to utilize acetaminophen and topical lidocaine for pain management throughout his inpatient rehabilitation admission.

#### 4.1.2. Pre- to Post-Intervention Effects

The participant reported increased average pain intensity during his hospitalization (Table 5). While the pain was initially musculoskeletal, over time it became more neuropathic. The participant noted an electrical shock quality to the pain in his neck and reported having painful spasms. He also reported increased pain interference as his pain increased. While pain intensity and interference increased, pain catastrophizing decreased, indicating that the participant had fewer negative automatic thoughts about his pain. Additionally, the participant’s willingness to engage in activities even when he experienced significant pain, as measured by pain acceptance, also increased. His sleep worsened significantly during his hospitalization.

#### 4.1.3. Clinical Presentation at Post-Intervention

Over his 53-day stay on the inpatient rehabilitation unit, the participant also received the standard of care Rehabilitation Psychology support. His psychologist (KSW), who was aware of his participation in the study, had a total of five visits with the participant, most of which occurred in the second half of the patient’s admission. Frequency of visits was tailored to meet the participant’s needs. Emotionally, the participant reported normative grief and worries about his prognosis, but he did not experience clinically significant symptoms of depression or acute stress disorder. He also did not experience symptoms of generalized anxiety disorder. However, over time the participant reported increased anxiety related to apneic events occurring more frequently during the night. Concurrently, he was evaluated by the Sleep Medicine consult service and was diagnosed with severe sleep apnea. The participant utilized nasal pillows and a CPAP machine during hospitalization. During these apneic events, he felt a sense of panic that was amplified by his memories of two frightening instances before he was fully weaned from the ventilator when he needed assistance managing secretions, but his call light malfunctioned.

The participant engaged well with psychological interventions and was successful in using pursed lip breathing to slow the breathing rate and count breaths. Once calm enough, he was able to initiate the self-hypnosis he learned in HYP-CT, which helped to further manage anxiety.

#### 4.1.4. Post-Discharge Follow-Up

After discharge from the hospital, the participant reported increased pain intensity. His pain transitioned from musculoskeletal to neuropathic pain, and he reported that pain experience was primarily neuropathic at post-discharge follow-up. He stated that he continued to experience painful spasms on a daily basis. He stated that pain interfered with his daily activities “quite a bit”. However, he reported increased self-efficacy in managing his pain during daily activities. His sleep quality also improved significantly. He reported he no longer used CPAP, but rather slept with his head elevated, which improved sleep quality. While not assessed formally, he continued to deny symptoms of depression, stating, “I think about the positive”. Unfortunately, he did not have access to the audio recordings to engage in hypnosis practice at home. However, he stated that at least once per week, he would “think about the elevator” (i.e., a visualization of deepening during hypnosis induction) as a strategy to help build relaxation when he was experiencing pain. At a later follow-up, he reported using the image of the elevator every time he needed to transfer using the lift, a painful experience that occurred approximately four times daily.

### 4.2. Perception of Change and Treatment Satisfaction

On the PGIC, the participant reported that he was “much improved” since the start of the study on both post-intervention and post-discharge assessments. On the BSW, he rated that he received “much benefit” from the intervention and was “very satisfied”. Additionally, he stated that he would be “very willing” to continue the therapy. In fact, at post-discharge follow-up, the participant inquired about initiating outpatient rehabilitation psychology treatment in order to receive further HYP-CT to address chronic pain. Additionally, he requested that audio recordings be sent again to his email address so he could restart home practice. Upon learning of the participant’s inability to access hypnosis recordings, the study team again sent the hypnosis recordings to patient’s primary caregiver in order to facilitate access.

The participant reported that the hypnosis inductions “gave me peace of mind”. He reported “[the intervention] helped me a lot”. He particularly noted that he liked the sense of relaxation that he got from hypnosis inductions. He noted that the skill of self-hypnosis was something that he used regularly, stating, “I have that information, and I can always use it”.

## 5. Discussion

The results of this case study provide additional support for the promise of early, hypnotic cognitive therapy for individuals with SCI experiencing pain or distress while dependent on mechanical ventilation. Although average pain intensity and pain interference did not improve for this patient, he reported increases in self-efficacy and pain acceptance, both important factors in the experience of pain. He did not experience any negative effects and thought that the intervention provided helpful support during his weaning from mechanical ventilation. Additionally, he discontinued opioid pain medications and reported actively using the strategies taught in the intervention. Upon discharge to home, the participant most commonly used his self-hypnosis practice in preparation for transfers with his lift, one of the most painful parts of his care. Over time, it appeared that the lift had become a naturally occurring cue that induced the hypnotic response. Finally, this early introduction to HYP-CT prompted the participant to re-engage with listening to the recording of the hypnosis induction for continued management of his chronic pain at home.

### 5.1. Challenges during the Intervention

Standard application of this intervention involves significant verbal collaboration with the person receiving the treatment to identify both helpful and unhelpful thoughts, feedback on hypnosis sessions, practice planning, and evaluating barriers to utilizing learned skills. For the first two sessions, the participant was unable to phonate due to mechanical ventilation, which limited his ability to engage in this collaborative process. Communication style was adjusted based on the patient’s needs. The provider offered lip reading and use of a communication board. Open-ended questions were rephrased to yes/no or multiple-choice format when needed. By the third session, the participant was able to voice two to three words at a time, and by the fourth session, he was able to communicate more freely.

Due to the high level of care and therapy needs, the participant was only available to meet with the study provider approximately once per week. Several attempts had to be made each week before a suitable time for a treatment session was found. Despite these scheduling challenges, the participant received four intervention sessions within five weeks.

### 5.2. Practice

The participant reported significant barriers to self-hypnosis practice, noting that he was very busy throughout his day and rarely had uninterrupted time to listen to recordings. Additionally, because of COVID-related hospital visitor restrictions, the participant did not have visitors at bedside during most of his hospitalization and did not have regular help accessing his phone or other audio devices.

While the participant stated that he was unable to utilize the audio recordings due to access issues, he reported that he was able to, and did, practice self-hypnosis regularly without the recording. He noted that he often used this strategy to prepare himself before staff offered turns in bed, which was a time when he reported experiencing the most pain. By Session 4, the participant reported that it was getting easier to allow himself to be hypnotized. He stated that as he listed to the psychologist start the induction, he would focus on her voice and “the next thing you know, I’m so relaxed”. Additionally, he was able to access this self-created relaxation during turns, even without a full hypnotic induction. He reports that he would take a deep breath, feel more relaxation and comfort and the “pain [during turns] is not as bad as it was before”.

### 5.3. Conclusions 

This case report, as well as our initial pilot study [46], confirms that it is practical to teach HYP-CT techniques to individuals with SCI during inpatient rehabilitation, even if those individuals are limited in their ability to engage in verbal exchanges due to mechanical ventilation needs. Offering psychological pain treatment during inpatient rehabilitation could serve as an avenue to increase accessibility to effective treatment that may reduce the negative impacts of pain, ultimately improving quality of life over the long-term for individuals with SCI. Development of interventions to address pain in SCI is a critical unmet need for this population, as SCI pain has generally been refractory to pain treatments [61,62,63]. As strictly biological interventions, such as pharmacology, continue to be limited, it is important to continue to develop and evaluate interventions from a biopsychosocial conceptualization of SCI pain. Future research should continue to evaluate the effectiveness of intervention on pain intensity, but should also include interventions to address other factors of pain, including cognitive/evaluative, affective/emotional, and behavioral elements as well as other conditions that can be related to pain following SCI, such as spasticity. We will continue to evaluate HYP-CT in an ongoing RCT focusing on effectiveness, mechanisms of change, and long-term impact of this intervention on pain and pain interference. Particularly for those individuals utilizing mechanical ventilation, it may be important to examine how HYP-CT impacts factors related to their respiratory function, such as respiratory rate or breathing quality. However, anecdotal reports such as this case study suggest HYP-CT has strong potential as a nonpharmacological treatment for early SCI-related pain.

## Figures and Tables

**Table 1:** Intervention schedule.

Session	Hospital Day	Topics Reviewed (30–40 min)	Hypnotic Induction (~20 min) *
1	27	Psychoeducation on cognitive model of pain	Increasing tolerance of ambiguity regarding pain and its impact
2	40	Thought worksheets to identify negative and positive automatic thoughts	Automatization of the process of altering pain-related catastrophizing and any other alarming or maladaptive cognitions into more reassuring and realistic cognitions
3	47	Reviewing and adjusting negative and positive automatic thoughts	Continued automatization of reassuring or more realistic cognitions Extrernalizing physical discomfort or unhelpful thoughts
4	55	Skill review Use of Motivational Interviewing to promote continued practice	Age progression hypnosis strategy based on strategy described by Moshe Torem

* Every in-session hypnosis induction was audio recorded and provided to the patient.

**Table 2:** Assessment schedule.

Measures		Timepoints			
Domain	Measure	Baseline	Intervention	Follow-Up	Post-Discharge
Pain intensity	Numerical Rating Scale (NRS) ranging from 0 (No pain) to 10 (Pain as bad as you can imagine)	X	X	X	X
Pain interference	Patient-Reported Outcomes Measurement Information System (PROMIS) Pain Interference (PI) Short Form	X		X	
	Single Pain Interference Item from Short Form Survey (SF-36)				X
Pain type	Spinal Cord Injury Pain Instrument (SCIPI)	X		X	X
Pain catastrophizing	University of Washington Concerns about Pain (UW-CAP)	X		X	
Pain self-efficacy	University of Washington Pain Related Self-Efficacy Scale (PRSE)	X		X	
Pain acceptance	Chronic Pain Acceptance Questionnaire- Revised (CPAQ-R)	X		X	
Depression	Patient Health Questionnaire-9 (PHQ-9)	X		X	
Sleep quality	PROMIS Sleep Disturbance Short Form	X		X	
Analgesic use	Medical chart review			X	X
Patient impression of change	Patient Global Impression of Change (PGIC)			X	X
Perceived benefit and satisfaction	Benefit, Satisfaction, and Willingness to Continue Treatment (BSW)			X	X
Skills practice	Patient report of number of times listening to recording or practicing self-hypnosis as well as amount of time spent on each practice		X	X	X
Relief during hypnosis	NRS ranging from 0 (No relief) to 10 (Complete relief)		X		

**Table 3:** Response to sessions.

	Pre-Session		Post-Session	
	Avg. Pain 24 h	Current Pain	Current Pain	Relief in Session
Session 1	8	8	6	4
Session 2	8	5	5	10
Session 3	6	6	3	10
Session 4	8	8	6	6

**Table 4:** Opioid use.

	Mean Daily Oxycodone (Range)
Prior to session 1	8.50 mg (15–5 mg)
Session 1–2	4.62 mg (15–0 mg)
Session 2–3	0.71 mg (5–0 mg)
Session 3–4	0.00 mg
Post Session 4	0.00 mg

**Table 5:** Assessment outcomes.

Variable Name	Measure	Baseline	Follow-Up	Post-Discharge
Average pain Intensity	NRS	4	6	8.5
Worst pain intensity	NRS	8	8	9
Pain interference	PROMIS	59.9	67.4	4/5 “quite a bit”
Pain catastrophizing	CAP	35.9	31.3	35.9
Depression	PHQ9	5.63	6	--
Pain acceptance	CPAQ-R Activity Engagement	36	41.8	--
	CPAQ-R Pain Willingness	29	29.3	--
Pain self-efficacy	PRSE	52.7	52.7	69.2
Sleep disturbance	PROMIS	33.1	60.4	28.9

## Data Availability

The datasets generated during and/or analyzed during the current study are available from the corresponding author on reasonable request.
